# Supplementary material for: Theoretical investigation of pre-symptomatic SARS-CoV-2 person-to-person transmission in households
Source: Sci Rep. 2021 Jul 14;11:14488. doi: 10.1038/s41598-021-93579-w (PMC8280150; doi:10.1038/s41598-021-93579-w)
Supplement: Supplementary file 1 — Supplementary Information. [file 41598_2021_93579_MOESM1_ESM.pdf]

SUPPLEMENTARY INFORMATION

Theoretical investigation of pre-symptomatic SARS-CoV-2 person-to-person transmission in households

Yehuda Arav, Ziv Klausner, Eyal Fattal

Sensitivity analysis

Some of the parameters' values were obtained from studies that also reported the range of these values. Therefore, we have performed an extensive sensitivity analysis to check the robustness of the results. Specifically, we have examined the sensitivity of the model's reconstruction of the serial interval distribution and the SAR for variations in the following parameters: dose response (Figure S1A), exposure time scale (Figure S1B), decay rate on surfaces (Figure S1C), surface area of the hand (Figure S1C), surface area touched (Figure S1D), the median viral load (Figure S1E), and the fomite to finger transfer efficiency (Figure S1F) . As seen, the model's results remains within the range of the values reported in the literature for the examined range of parameters.

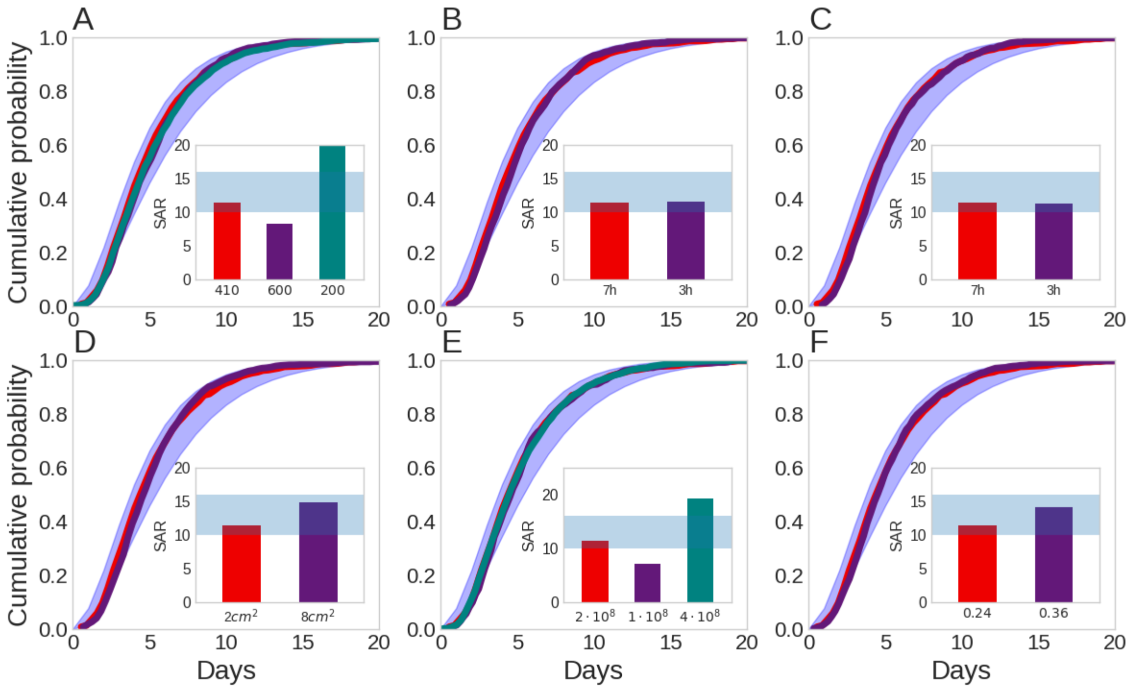

Figure S1. The prediction of the serial interval distribution and SAR (inset) for different parameter values. (A) dose response parameter (B) Exposure time scale (C) surface decay rate (D) surface area of a touch (E) median viral load. (F) fomite to finger transfer efficiency. We have maintained the ratio between the fomite to finger and finger to fomite transfer coefficients. Red bar represents the reference simulation.

We note that since the contribution of transmission modes 3 and 4 (Indirect contact transmission via surface and droplet nuceli transmission, respectively) is very small, the model is not sensitive to the parameters that are associated with them (such as the area of the room, the decay rate in air, breathing rate and etc.).
